# LymphGen基因分型在弥漫大B细胞淋巴瘤患者中的分布特征及预后价值验证

**DOI:** 10.3760/cma.j.issn.0253-2727.2022.04.007

**Published:** 2022-04

**Authors:** 芳 张, 阿不来提 热那古力, 小龙 漆, 珍 寇, 顺生 翟, 巍 谭, 阿布都尔 木合拜尔, 玉玲 聂, 燕 李

**Affiliations:** 1 石河子大学医学院，石河子 832001 Medical School of Shihezi University, Shihezi 832001, China; 2 新疆维吾尔自治区人民医院血液科，乌鲁木齐 830001 Department of Haematology, People's Hospital of Xinjiang Uygur Autonomous Region, Urumqi 830001, China

**Keywords:** 淋巴瘤，大B细胞，弥漫性, LymphGen基因分型, 二代测序, 分布, 预后, Lymphoma, large B-cell, diffuse, LymphGen genotyping, Next generation sequencing, Distribution, Prognosis

## Abstract

**目的:**

了解LymphGen基因分型在弥漫大B细胞淋巴瘤（DLBCL）人群中的分布特征并验证其预后价值。

**方法:**

收集2014年6月至2020年12月在新疆维吾尔自治区人民医院资料完整155例初诊DLBCL患者的临床资料及石蜡包埋肿瘤组织标本，从肿瘤组织中提取DNA，应用二代测序技术检测475种基因突变情况，研究LymphGen基因分型在新疆DLBCL人群及不同细胞起源（COO）分型患者中的分布情况及对无进展生存（PFS）及总生存（OS）的影响。

**结果:**

①155患者中 105例（67.7％）能进行基因分型，其中MCD型14例（9.0％），BN2型26例（16.8％），N1型10例（6.5％），EZB型8例（5.2％），A53型27例（17.4％），ST2型20例（12.9％）。②各基因亚型在不同COO分型中分布不同（*P*＝0.021），生发中心型（GCB）组中以ST2为主（28.8％），非生发中心型（non-GCB）组中以A53及MCD型为主（35.8％、17.0％），BN2型在两组中均较多分布（23.1％、26.4％）。③不同基因亚型PFS、OS差异有统计学意义（*P*值分别为0.031、0.005）：N1型2年PFS、OS率仅为（21.3±18.4）％，A53型3年PFS、OS率分别为（60.9±11.3）％、（46.8±10.9）％。④MCD型3年PFS、OS率最优，但5年PFS、OS率较差。⑤LymphGen基因分型预测OS的受试者工作特征曲线（ROC曲线）曲线下面积为0.66，具有一定的区分度。

**结论:**

LymphGen基因分型在DLBCL人群中分布与以往报道有差异，LymphGen基因分型对DLBCL患者预后判断有一定价值。

## Abstract (en)

**Objective:**

To investigate the distribution characteristics of LymphGen genotyping in a diffuse large B-cell lymphoma（DLBCL）population and verify its prognostic value.

**Methods:**

We collected the clinical data and paraffin-embedded tumor tissue samples of 155 patients with newly diagnosed DLBCL in the People's Hospital of Xinjiang Uygur Autonomous Region from June 2014 to December 2020. DNA was extracted from tumor tissue and 475 gene mutations were detected by next-generation sequencing technology. We investigated the distribution of LymphGen genotyping in the DLBCL population, patients with different COO genotypes in the Xinjiang region, and their effects on PFS and OS.

**Results:**

①Among 155 patients, 105 patients（67.7％）could be genotyped, including 14（9.0％）for MCD, 26（16.8％）for BN2, 10（6.5％）for N1, 8（5.2％）for EZB, 27（17.4％）for A53, and 20（12.9％）for ST2. ②The distribution of each gene subtype was different in different cell origin（COO）types（*P*＝0.021）. ST2 was dominant in the germinal center type（GCB）group（28.8％）, and A53 and MCD were dominant in the non-GCB group（35.8％, 17.0％）. The BN2 type was the most common in both groups（23.1％, 26.4％）. ③There were statistically significant differences in progression-free survival（PFS）and overall survival（OS）among different gene subtypes（*P*＝0.031 and 0.005, respectively）. N1 and A53 had poor prognosis. The 2-year PFS and OS rates of N1 were both（21.3±18.4）％, and the 3-year PFS and OS rates of A53 were（60.9±11.3）％,（46.8±10.9）％, respectively. ④The 3-year PFS and OS rates of MCD were the best, but the 5-year PFS and OS rates were worse. ⑤In the ROC curve of LymphGen genotyping for OS prediction, the AUC was 0.66, showing a certain degree of differentiation.

**Conclusion:**

LymphGen genotyping in the DLBCL population was different from previous reports and was of great significance for the prognosis of patients with DLBCL.

弥漫大B细胞淋巴瘤（DLBCL）是成人淋巴瘤中最常见的一种类型。在中国，DLBCL占所有非霍奇金淋巴瘤（NHL）的45.8％，占所有淋巴瘤的40.1％[1]。免疫化疗的应用使DLBCL患者的预后得以改善，但仍有30％～40％的患者表现为难治或复发，主要原因是DLBCL在临床、免疫表型及分子遗传学等方面具有高度异质性[2]。二代测序（NGS）是一种新型基因检测技术，具有通量高、灵敏度高、可定量和成本低的优势[3]。近年来，不断有研究者应用NGS技术进一步完善DLBCL的基因分型，2020年Wright等[4]利用NGS技术提出了LymphGen基因分型，可以从分子遗传学层面更加精准评估DLBCL患者的预后。目前LymphGen基因分型在国内DLBCL患者中的预后价值尚未得到验证，因此，在本研究中我们应用NGS检测155例初诊DLBCL患者石蜡包埋肿瘤组织的基因突变情况，探索LymphGen基因分型在DLBCL人群中的分布情况并验证其预后价值。

## 病例与方法

1. 病例资料：回顾性分析2014年6月至2020年12月在新疆维吾尔自治区人民医院确诊且留存石蜡包埋肿瘤组织标本的初诊DLBCL患者临床资料。诊断标准参照《中国弥漫大B细胞淋巴瘤诊断与治疗指南（2013年版）》[5]，所有患者确诊前未接受任何放、化疗，未合并其他血液系统肿瘤和（或）恶性消耗性疾病，临床及随访资料完整。收集患者年龄、性别、乳酸脱氢酶（LDH）、病理组织学、免疫组化、影像学及骨髓检查等资料，分期采用 Ann Arbor分期，预后评估采用国际预后指数（IPI）评分，细胞起源（COO）分型采用Hans分型方法。本研究获新疆维吾尔自治区人民医院伦理委员会批准（ky2020041041）。

2. LymphGen基因分型定义：N1型（NOTCH1突变），MCD型（MYD88L265P、CD79B突变），BN2型（NOTCH2突变或BCL6易位），EZB型（EZH2突变或BCL2融合），A53型（非整倍体的TP53失活）和ST2型（SGK1、TET2突变），其余为Others[4]。

3. NGS测序：对全部患者石蜡包埋肿瘤组织标本进行DNA提取，由南京世和基因生物技术股份有限公司采用安捷伦SureSelect Human All Exon Kit（美国Santa Clara公司产品）的靶向捕获方法进行测序，基因PANEL包括475种热点基因，然后在Illumina HiSeq平台对富集片段进行大规模并行测序，20×靶区覆盖率>90％，使用SNV/small indel完成检测、注释及统计。

4. 荧光原位杂交（FISH）检测及结果判读：经NGS检测确定为EZB型的患者通过FISH检测MYC重排，选取合适蜡块制作组织芯片，4 µm连续切片，按照MYC双色分离探针试剂盒（美国雅培 Visis 公司产品）说明书进行操作。结果判读：正常细胞显示2个红绿融合信号，基因易位阳性细胞显示1个红绿融合信号及2个分离的红绿信号。每个探针检测至少计数200个细胞，出现3个及3个以上阳性细胞判断为阳性。

5. 治疗方案：155 例初治DLBCL患者均采用标准R-CHOP方案（利妥昔单抗+环磷酰胺+表柔比星+长春新碱+泼尼松）6～8个疗程，其中23例进行自体造血干细胞移植巩固治疗。二线方案包括顺铂+阿糖胞苷+地塞米松（DHAP）、异环磷酰胺+卡铂+依托泊苷（ICE）和吉西他滨+地塞米松+顺铂（GDP）方案。

6. 随访：通过查阅病历资料及电话进行随访，随访截止日期为2021年6月。所有患者均进行了相应影像学（CT、MRI或PET/CT）的疗效评价，疗效评价包括完全缓解（CR）、部分缓解（PR）、疾病稳定（SD）、复发或进展（PD）。无进展生存（PFS）期定义为从治疗开始至首次疾病进展或随访截止的时间；总生存（OS）期定义为从治疗开始至任何原因导致的死亡或随访截止的时间。

7. 统计学处理：采用SPSS 26.0及R 3.5.3进行统计学分析，ComplexHeatmap包绘制热图。其中分类变量用百分率（％）表示，组间比较采用Fisher精确概率法。通过Kaplan-Meier法绘制生存曲线，Log-rank检验进行组间比较，*P*<0.05为差异有统计学意义。绘制受试者工作特征曲线（ROC曲线）评估LymphGen基因分型对生存的预测准确度，曲线下面积（AUC）>0.5时评估分型具有预测价值，AUC 值0.5～0.75时认为准确性一般，>0.75时认为准确性较好。

## 结果

1. LymphGen基因分型在DLBCL中的分布情况：共155例DLBCL患者纳入研究，参照LymphGen基因分型方法进行基因分型，基因突变图谱见图1，其中MCD型14例（9.0％），BN2型26例（16.8％），N1型10例（6.5％），EZB型8例（5.2％），A53型27例（17.4％），ST2型20例（12.9％），Others 50例（32.3％）。因本研究仅2例EZB型FISH检测MYC阳性，故未对EZB型进行进一步分型。

**图1:**
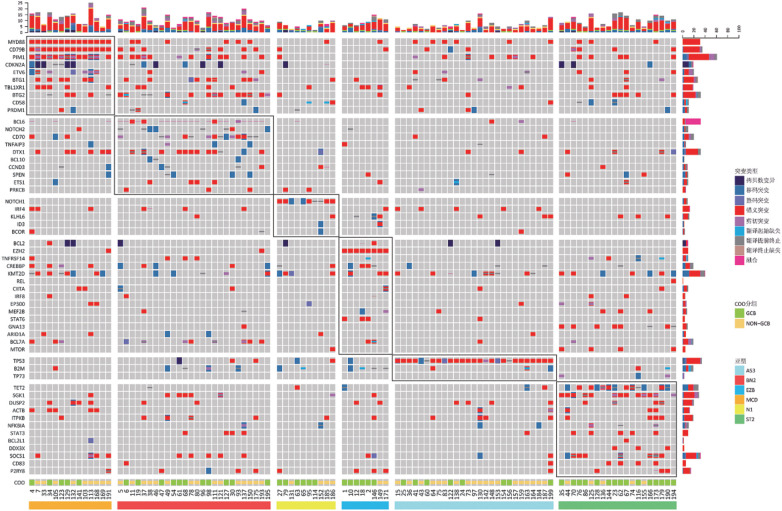
105例弥漫大B细胞淋巴瘤患者LymphGen基因分型突变图谱 COO：细胞起源；GCB：生发中心型；non-GCB：非生发中心型

LymphGen基因分型可明确分型的患者共105例（67.7％），临床特征见表1。其中，男55例，女50例；>60岁58例，≤60岁47例；经Hans分型GCB型52例，non-GCB型53例；Ann Arbor分期Ⅰ、Ⅱ期28例，Ⅲ、Ⅳ期77例；IPI评分相对低危55例，相对高危50例。不同年龄（*P*＝0.935）、性别（*P*＝0.414）、LDH水平分组（*P*＝0.268）、原发部位（*P*＝0.380）、受累部位（*P*＝0.198）、Ann Arbor分期（*P*＝0.629）和IPI评分（*P*＝0.659）各基因亚型分布差异均无统计学意义。但是，GCB与non-GCB两组基因亚型分布差异有统计学意义（*P*＝0.021），GCB组中BN2、ST2更常见，non-GCB组中A53比例较高。

**表1:** 105例LymphGen基因分型弥漫大B细胞淋巴瘤患者基因分型与临床特征的关系［例（％）］

临床特征	例数	LymphGen基因亚型						*P*值
		MCD	BN2	N1	EZB	A53	ST2	
年龄								0.935
≤60岁	47	5（10.6）	12（25.5）	4（8.5）	4（8.5）	14（29.8）	8（17.0）	
>60岁	58	9（15.5）	14（24.1）	6（10.3）	4（6.9）	13（22.4）	12（20.7）	
性别								0.414
男	55	11（19.6）	12（21.4）	5（8.9）	5（8.9）	14（25.0）	9（16.1）	
女	50	3（6.1）	14（28.6）	5（10.2）	3（6.1）	13（26.5）	11（22.4）	
细胞起源分型								0.021
GCB	52	5（9.6）	12（23.1）	6（11.5）	6（11.5）	8（15.4）	15（28.8）	
non-GCB	53	9（17.0）	14（26.4）	4（7.5）	2（3.8）	19（35.8）	5（9.4）	
LDH								0.268
<245 U/L	58	11（19.0）	11（19.0）	5（8.6）	5（8.6）	13（22.4）	13（22.4）	
≥245 U/L	47	3（6.4）	15（31.9）	5（10.6）	3（6.4）	14（29.8）	7（14.9）	
原发部位								0.380
结内	64	8（12.5）	18（28.1）	7（10.9）	2（3.1）	17（26.6）	12（18.8）	
结外	41	6（14.6）	8（19.5）	3（7.3）	6（14.6）	10（24.4）	8（19.5）	
受累部位								0.198
结内	54	5（9.3）	16（29.6）	6（11.1）	2（3.7）	17（31.5）	8（14.8）	
结外	51	9（17.6）	10（19.6）	4（7.8）	6（11.8）	10（19.6）	12（23.5）	
Ann Arbor分期								0.629
Ⅰ、Ⅱ期	28	4（14.3）	10（35.7）	3（10.7）	1（3.6）	5（17.9）	5（17.9）	
Ⅲ、Ⅳ期	77	10（13.0）	16（20.8）	7（9.1）	7（9.1）	22（28.6）	15（19.5）	
IPI评分								0.659
0～2分	55	6（10.9）	15（27.3）	5（9.1）	4（7.3）	17（30.9）	8（14.5）	
3～5分	50	8（16.0）	11（22.0）	5（10.0）	4（8.0）	10（20.0）	12（24.0）	

注：GCB：生发中心型；non-GCB：非生发中心型；IPI：国际预后指数

2. LymphGen基因分型的预后价值：截至2021年6月，105例明确LymphGen基因分型患者中位随访20（3～82）个月，29例（27.6％）因肿瘤复发或进展死亡，其中N1型5例，A53型11例，占比较高。Kaplan-Meier生存分析结果显示各基因亚型间5年PFS、OS率差异有统计学意义（*P*值分别为0.031、0.005）（图2）。其中，N1、A53型预后较差，两组人群因死亡例数较多未随访到5年，其中N1型2年PFS、OS率仅为（21.3±18.4）％，为生存最差亚型，A53型3年PFS、OS率分别为（60.9±11.3）％、（46.8±10.9）％。其余亚型预后较好，其中BN2型5年PFS、OS率分别为（66.3±8.8）％、（74.9±5.9）％，MCD型5年PFS、OS率分别为（42.2±21.7）％、（69.6±20.8）％，ST2型5年PFS、OS率分别为（72.7±12.3）％、（79.2±11.0）％，EZB型5年PFS、OS率分别为（60.0±18.2）％、（75.0±15.3）％。MCD型3年PFS、OS率最优，但5年PFS、OS率较差。

**图2:**
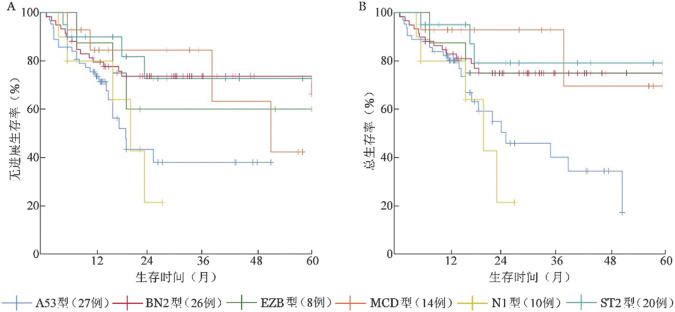
105例不同LymphGen基因分型弥漫大B细胞淋巴瘤患者的无进展生存（A）及总生存（B）曲线

绘制不同LymphGen基因分型预测OS的ROC曲线（图3），AUC＝0.66，提示LymphGen基因分型评估本组105例DLBCL患者生存风险具有一定的区分度，LymphGen基因分型在本组DLBCL患者预后评估中具有一定价值。

**图3:**
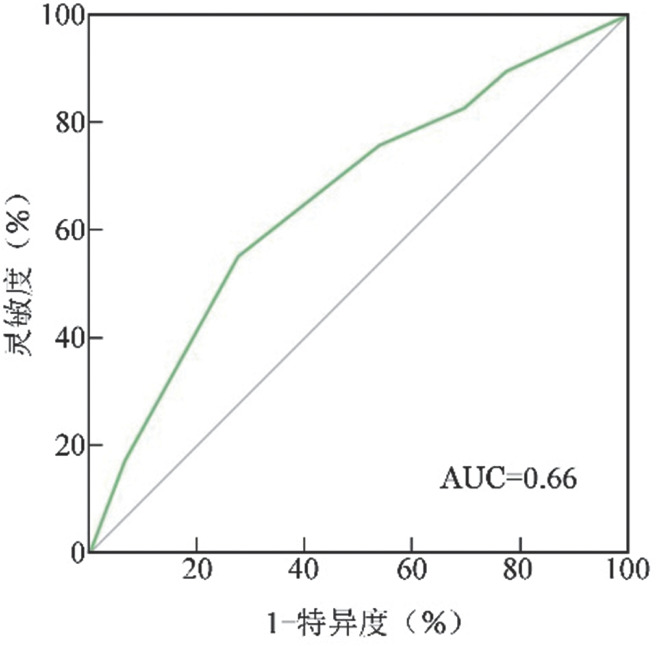
LymphGen基因分型评估弥漫大B细胞淋巴瘤患者生存风险的受试者工作特征（ROC）曲线 AUC：曲线下面积

## 讨论

近年来，基于NGS检测DLBCL基因突变情况倍受关注，2017年Reddy等 [6]利用NGS检测确定了150个基因在DLBCL中的驱动作用。2018年Schmitz等[7]提出了新基因分型（MCD、BN2、N1、EZB型），这4种亚型细胞起源和对免疫治疗的反应不同，该研究结果对于DLBCL的精准治疗具有里程碑的意义，但仅有46.6％患者可以进行具体的分型。2020年Wright等[4]提出了“LymphGen基因分型”，在4分型的基础上加入了A53型和ST2型，并将EZB型进一步分为MYC^+^和MYC^−^两种亚型，可将纳入分型患者的比例提高至63.1％，是目前最全面的DLBCL基因分型。

本研究155例DLBCL患者中可进行LymphGen基因分型患者占67.7％，与Wright等[4]报道63.1％相近，但分析各亚型的分布比例，其中A53型（17.4％）最多，同国内（16％）报道一致，但显著高于国外（6.6％）；而本研究中MCD型（9.0％）明显低于国外Wright等[4]、国内Zhang等[8]报道（13.9％、20％），与Schmitz等[7]报道（8.0％）一致；EZB型（5.2％）与国内报道（2％）接近，显著低于国外报道（13.2％）；N1型（6.5％）与ST2型（12.9％）均高于国外[4],[7]报道；BN2型与国内外报道一致，上述研究结果表明DLBCL基因分型可能在不同国家、不同地区存在差异。

本研究还比较了各分型不同年龄、性别、LDH水平、原发及受累部位、Ann Arbor分期和IPI等临床因素的差异性，仅在COO分型之间有差异。GCB中以ST2较多分布（28.8％），non-GCB组中以A53较多分布（35.8％），这与文献[4],[7]报道GCB以EZB型为主、non-GCB中以MCD分布为主不同。BN2型在两组中均较多分布（23.1％、26.4％），这与文献[4],[7]报道相似，表明基因分型在不同国家分布特征有差异。该研究结果提示不同国家、不同人种DLBCL可能存在细胞遗传学差异。此外，国内多数报道认为non-GCB比例较高[9]–[10]，而本组病例中GCB与non-GCB比例相近，与国内报道有一定差异，这也是导致本研究基因分型分布与国内差异的另一个重要原因。另外，基因分型的差异性是否与本研究中民族结构差异有关，因病例数有限未能进一步分组比较，需要进一步积累病例研究证实。

多项研究表明[11]–[12]，TP53突变和17p/TP53缺失的DLBCL患者较差的预后相关，N1以获得NOTCH1突变为特征，在DLBCL中NOTCH1突变亦提示较差的预后，本研究对基因分型进行生存分析时，显示A53及N1型PFS、OS均差于其他亚型，而ST2型与BN2型预后较好，与文献[4],[7]报道相符。MYD88突变在non-GCB DLBCL中更普遍且预后差[11]–[13]，MYD88经常与CD79B突变相关联[4],[7],[14]，因此 MYD88和CD79B突变标志着DLBCL的不良预后。本研究中MCD型的3年OS率较好，与文献[4],[7]报道不同，但5年OS率较3年OS率明显下降，这可能与本研究中多数MCD亚型患者随访时间较短尚未发生结局事件有关，这仍提示MCD为一种较差的预后基因分型，需后续继续随访验证。另外本研究因EZB病例数较少且仅2例MYC重排阳性，未将EZB进一步分组，这使得EZB型的生存具有一定的病例数限制与分组干扰，需后续扩大样本量，纳入更高比例的分型并结合FISH结果进行分析。

最后，本研究通过ROC曲线对基因分型在本研究队列中的区分度进行评估，AUC为0.66，具有一定的区分度，但与Schmitz等[7]及Wright等[4]研究结果相比，区分度较差。这可能是由于本研究病例数较少、基因分型分组较多、且属于回顾性研究有关。本研究病例包含了新疆各地区的DLBCL人群，具有一定的代表性，在一定程度反映新疆地区DLBCL基因分型的分布及预后情况。

综上所述，本研究应用NGS探究初诊DLBCL患者基因型分布情况，证实不同LymphGen基因分型人群预后不同。本研究部分结果与既往文献报道不同，提示DLBCL基因亚型可能与地域、民族有关。
